# Rational Design of a Cu(II) Spin Label Improves the Sensitivity of Distance Measurements

**DOI:** 10.1021/acs.jpclett.5c02221

**Published:** 2025-09-26

**Authors:** Shramana Palit, Zikri Hasanbasri, Nicholas A. Moriglioni, Joshua Casto, Sunil Saxena

**Affiliations:** Department of Chemistry, 6614 University of Pittsburgh, 219 Parkman Avenue, Pittsburgh, Pennsylvania 15260, United States

## Abstract

The measurement of distance constraints in biomolecules using Cu­(II) spin labels at Q-band EPR is complicated due to the selective excitation of only some orientations of the label. Here, we introduce a new Cu­(II) spin label, in which the divalent metal is coordinated equatorially by four nitrogen atoms. Such chelation reduces the *g* anisotropy of the Cu­(II) spin, which reduces the spectral width. We show that the complex coordinates to target labeling sites on a protein and provides narrow distance distributions on proteins. DEER data acquired across different fields at Q-band show the same period of modulation, which suggests proper orientational averaging. Thus, a single measurement at the magnetic field corresponding to the maximum intensity is sufficient to measure the distance distribution. Although the label does not bind stoichiometrically, we observe a 2.3-fold improvement in sensitivity in half the data acquisition time compared to those of existing Cu­(II) spin labels.

## Graphical abstract

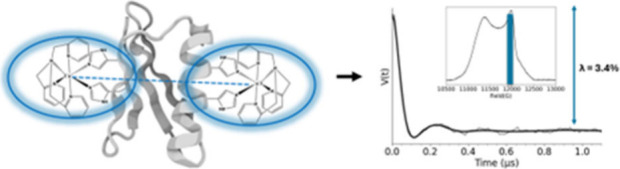

Electron paramagnetic resonance (EPR) in combination with site-directed spin labeling^1,2^ has emerged as a powerful technique to study biomolecular dynamics and conformations. In this methodology, spin labels are incorporated site specifically in a protein. Continuous wave (CW) EPR experiments are then used to study site-specific dynamics as the spectral line shapes are sensitive to small-amplitude fluctuations of the protein backbone.^3–7^ Furthermore, pulsed dipolar EPR^8–12^ methods on doubly labeled biomolecules provide structural information via measurement of distance constraints. Thus far, organic radicals like nitroxide^1,13–19^ and trityl^20–22^ and paramagnetic metal ions like Gd­(III)^23–25^ and Cu­(II)^26–29^ have been effectively used as spin labels for biomolecules. Such a site-directed methodology was initially popularized by spin labels that attach to a cysteine residue in a protein via a disulfide linker. However, these cysteine labeling methods have some limitations. The linker that connects the paramagnetic moiety to the protein backbone has rotatable bonds, which impart an inherent rotameric flexibility to the label. This flexibility of the linker results in broader distance distributions, which makes the interpretation of distance information in terms of small conformational change or protein structure difficult.^30,31^ This limitation has motivated the development of bipedal nitroxide spin labels that involve chelation to two cysteines^17^ and rigid linkers.^32–35^ Another drawback with cysteine labeling is that native accessible cysteines must be removed, which can be problematic in some cases. This has led to the advent of labels that react with tyrosine, methionine, and lysine residues and spin labeling through noncanonical amino acids.^36–39^

In order to reduce the contributions of the label to the distribution, Cu­(II)-based spin labels for proteins have recently been developed.^40,41^ The labeling scheme utilizes two strategically mutated histidine residues on the protein to bind to the Cu­(II) label.^41^ This bipedal attachment imparts a high rigidity to the label. In the double-histidine (dHis) motif, the label binds to *i*, *i* + 4 and *i*, *i* + 2 arrangements of histidine residues on α-helical and β-sheet sites, respectively. Moreover, the label utilizes Cu­(II) complexed with either iminodiacetic acid (IDA) or nitrilotriacetic acid (NTA) in order to enhance specificity of binding to dHis sites and to eliminate nonspecific binding to other sites of the protein, including single native His sites.^40–45^

Distance measurements using this dHis-Cu­(II) can provide distance distributions that are up to 5 times narrower compared to commonly used nitroxide spin labels,^41^ leading to new pathways for the use of spin labels for sensitive measurements^46,47^ of protein structure and conformational dynamics in vitro^45,48,49^ and in cells^50–52^ in EPR and beyond.^53–56^ On the other hand, Cu­(II)-based spin labels have a broader spectral width compared to that of nitroxides, which presents two main challenges in EPR. First, a narrow bandwidth pulse is unable to efficiently excite a large portion of the spectrum, leading to the loss of signal sensitivity in distance measurements. The loss of sensitivity for Cu­(II) is ameliorated to some extent by deuteration to enhance phase memory time,^57^ operation at lower temperatures, which enhances spin polarization,^58^ and the use of frequency-swept chirp pulses to enhance spectral excitation.^59^ Typically, Cu­(II)-based distances are measured with concentrations 2–5 times higher than those of nitroxide-based measurements. Moreover, with Cu­(II) labels, distances of up to ca. 7 nm have been measured, although this range can be extended with deuteration.^57^ For nitroxide, distance measurements of ca. 16 nm have been achieved with full protein deuteration.^60,61^ Second, at Q-band frequencies, only some orientations of the interspin vector are excited at a given magnetic field, resulting in a phenomenon called orientational selectivity. In effect, the extraction of distance information from the dipolar signal measured at a given magnetic field requires knowledge of the relative orientation of the two spins that are excited, which is unknown for a given sample. Therefore, several experiments across the EPR spectrum must be performed to statistically sample all orientations, which extends the experimental data collection times.^49,62–64^ Thus, there is a need for new approaches to circumvent orientational effects at Q-band for Cu­(II)-based distance measurements.

In this work, we explore a chemical approach to alleviate orientation selection in pulsed dipolar spectroscopy. We reexamined the chelation strategy of Cu­(II). In Cu­(II)-NTA, the divalent metal cation is directly coordinated to three oxygens and one nitrogen. We hypothesized that replacing existing directly coordinated oxygen atoms with nitrogen would lead to a reduction of *g* anisotropy.^65,66^ Nitrogen atoms donate electrons and thus increase the electron density near the Cu­(II) nucleus. The consequent change in spin orbit coupling results in a decrease in *g* anisotropy and spectral width, which improves pulse excitation. Here, we report a new Cu­(II) complex as a spin label for proteins, where Cu­(II) is chelated to tris­(2-pyridylmethyl)­amine (TPA). In this work, we show that Cu­(II)-TPA rigidly coordinates to dHis and accurately measures protein backbone distances. Furthermore, we also show that DEER experiments at the Q-band do not exhibit orientational selectivity with the new label. Additionally, there is a ca. 4-fold improvement in sensitivity in comparison to Cu­(II)-NTA.

We prepared the copper­(II) tris­(2-pyridylmethyl)­amine (Cu­(II)-TPA) complex according to a previously reported protocol.^67^ Continuous wave (CW) EPR was first performed to ascertain the labeling. Figure 1A shows the CW-EPR spectra of free CuCl_2_ (gray) and the Cu­(II)-TPA spin label (black) in HEPES buffer at pH 7.4. The corresponding structures are shown at the left. There is a distinct spectral shift between the CW spectrum of CuCl_2_ and that of Cu­(II)-TPA, which indicates that Cu­(II) has undergone complete complexation with the TPA ligand. For the complex, the *g*_*yy*_, *A*_*yy*_, and *A*_*zz*_ regions of the spectrum were not well resolved at X-band (cf. Figure 1A), making accurate simulations difficult. Therefore, we acquired the field-swept electron spin echo-detected spectrum (FS-ESE) of free Cu­(II)-TPA at Q-band (cf. Figure S1B), which helped resolve the *g*_*zz*_ and *A*_*zz*_ portion of the spectrum. Moreover, the X-band and Q-band data (cf. Figure S1) taken together show that the spectrum for Cu­(II)-TPA has the following values: *g*_*xx*_ = 2.199, *g*_*zz*_ = 2.030, *A*_*xx*_ = 113 G, and *A*_*zz*_ = 98 G. Although *g*_*yy*_ and *A*_*yy*_ were poorly resolved even at Q-band, we estimated *g*_*yy*_ to be around 2.188 and *A_yy_* to be around 75 G. The values of **g** and **A** tensors are indicators of the coordination geometry, and for Cu­(II)-TPA, we found that *g_xx_* > *g*_*yy*_ > *g*_*zz*_. This trend is indicative of an intermediate trigonal bipyramidal/square planar (tbp/sp) geometry.^68–71^

**Figure 1:**
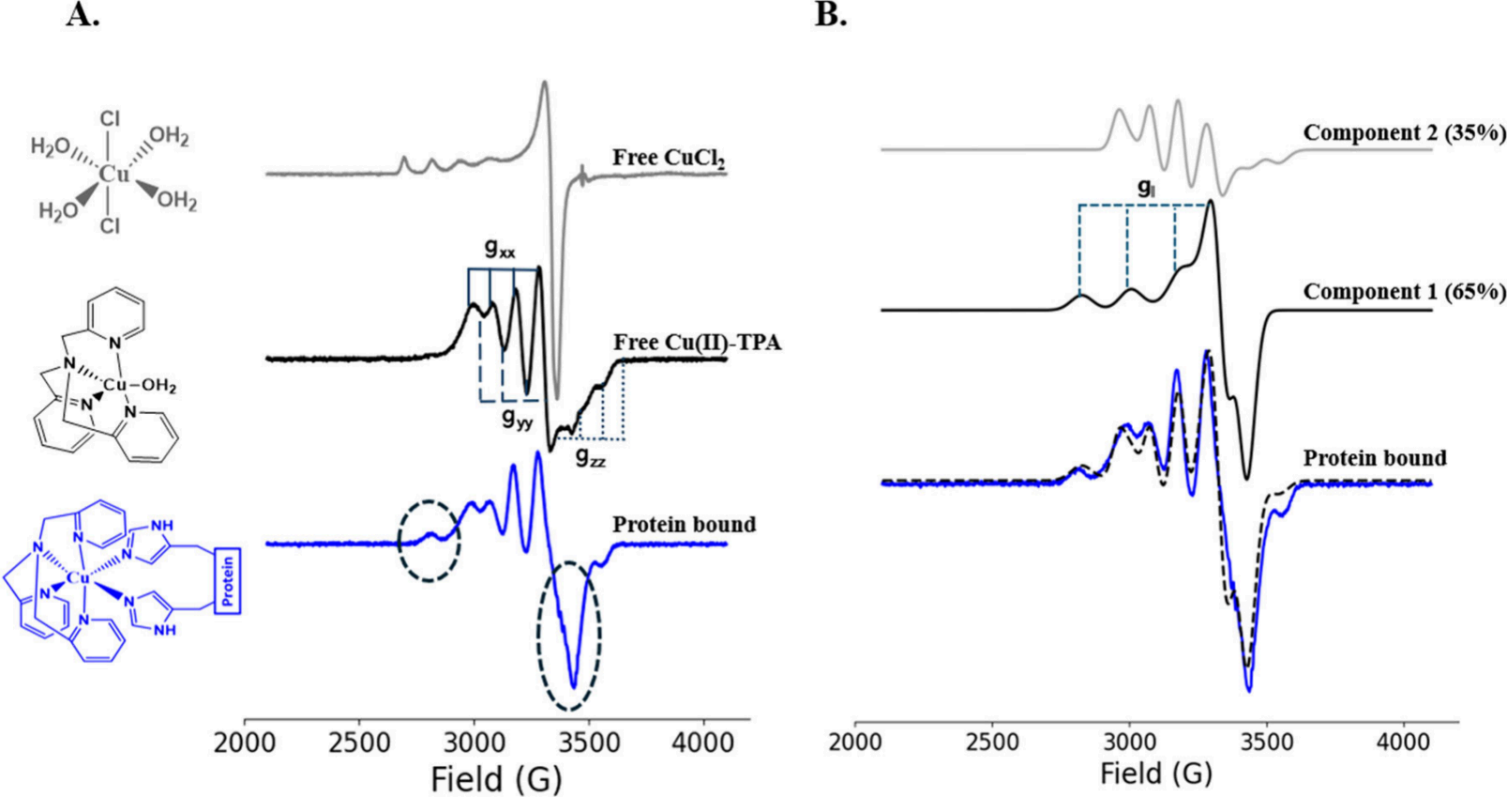
(A) CW-EPR spectra of CuCl_2_ (gray), Cu­(II)-TPA in HEPES buffer at pH 7.4 (black), and Cu­(II)-TPA bound to tetramutant 15H/17H/28H/32H GB1 (blue). The respective molecular structures are shown at the left. For free Cu­(II)­TPA, the rhombic **g** tensors are indicated on the spectrum. (B) Experimental (blue) and simulated (black dashed) CW-EPR spectra of dHis-bound Cu­(II)-TPA. Component 1 (65%) is indicative of an octahedral coordination arising from Cu­(II)-TPA coordinating to two histidine sites on the protein shown in black. The second component is identical to free Cu­(II)-TPA in solution (cf. spectrum in light gray).

To determine the conditions for the binding of the label to dHis sites, we prepared four dHis mutants of the B1 immunoglobulin binding domain of protein G (GB1):^72^ E15H/T17H with dHis on a β-sheet, K28H/Q32H with a dHis site on an α-helix, and two tetramutants (E15H/T17H/K28H/Q32H and I6H/N8H/K28H/Q32H)^40–42^ with dHis sites on a β-sheet and an α-helix. The structure and dynamics of GB1 protein have been thoroughly characterized by crystallography,^72^ NMR,^73,74^ and EPR.^48,75,76^ In addition, the stability of dHis mutants has been measured previously through circular dichroism.^41^ Therefore, the protein serves as an important well-calibrated system for this work. Next, we determined the loading efficiency of the dHis protein on Cu­(II)-TPA by CW-EPR spectroscopy. For successful coordination to dHis of the protein, the Cu­(II) complex should transition from a five-coordinate trigonal planar complex to a six-coordinate octahedral geometry (cf. structure of protein-bound Cu­(II)-TPA shown in the bottom panel of Figure 1A).

Figure 1A shows the CW-EPR spectrum of GB1-bound Cu­(II)-TPA in HEPES buffer at pH 7.4 (cf. bottom trace in blue). There are significant changes in spectral line shape when the label binds to protein, which are evident specifically around 2800 and 2450 G. These regions are highlighted by dashed ellipses in Figure 1A. The spectrum can be simulated with two components as shown in Figure 1B. The predominant component 1 (65 ± 5%) has **g** and **A** tensors that are characteristic of octahedral coordination.^68^ The values of *g*_∥_ and *A*_∥_ of component 1 are 2.231 and 171 G, respectively, and are in the range of four nitrogen atoms coordinating to the Cu­(II) center in the equatorial plane.^65^ The **g** and **A** tensors of component 2 have the same values as those of free Cu­(II)-TPA in a buffer. The dominant octahedral component suggests that the label coordinates to the dHis sites on a protein. The values of the **g** and **A** tensors of the bound and free label are listed in Table S1. CW-ESR data of 28H/32H GB1 and 15H/17H GB1 were also measured, and these data are shown in Figure S2. The simulations indicate that the percentage of the bound component is 80 ± 5% for the α-helical site and 70 ± 3% for the β-sheet site. Based on these values, the overall labeling efficiency of doubly bound GB1 is expected to be 56 ± 4%.

Next, we tried to optimize the labeling conditions by adding stoichiometric amounts of the label to E15H/T17H/K28H/Q32H GB1. We used HEPES buffer under two pH conditions, 6.5 and 7.4. At both values of pH, labeling was performed with and without a cosolvent (acetonitrile). The solubility of TPA in buffer was a challenge due to its hydrophobicity, and acetonitrile improved the solubility. We performed CW-EPR experiments, and simulated spectra to ascertain the percent of bound label. We found that the labeling efficiency was higher at pH 7.4 (∼65–70%) than at pH 6.5 (∼55%). These data are shown in Figure S3A. This result is expected, as a slightly basic pH is favored for the deprotonation of the nitrogen of the imidazole rings that bind to the Cu­(II) center. The use of a cosolvent had a minimal impact on protein labeling. Therefore, all of the data presented in the text were collected without the use of any cosolvents.

Next, we performed CW experiments at different time increments of incubation to determine the optimal time of incubation of the protein with the spin label. The spectra obtained were then simulated to obtain the percent of dHis binding at each time step. Figure 2A shows the amount of the octahedral component as a function of incubation time. The primary CW-EPR spectra are shown in Figure S4. The optimal labeling is achieved in ca. 10 min, which is faster than Cu­(II)-NTA where optimal labeling requires incubation for ∼30 min.^77^

**Figure 2:**
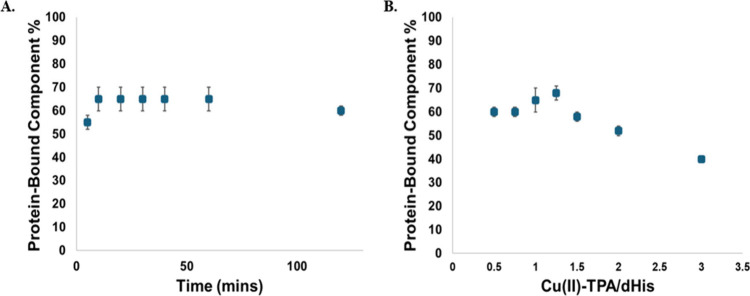
(A) The variation in percent of protein labeling versus incubation time is shown. The CW-EPR spectrum for each incubation time was simulated to generate the percent of bound label. (B) The labeling efficiency as a function of the Cu­(II)-TPA:dHis ratio is shown. The ratio of 1.25 times label per histidine site is optimal for labeling with Cu­(II)-TPA. The error bars are calculated by changing the component ratio in EasySpin until the simulated spectrum changes significantly.

The incubation temperature is an important factor to consider, as the reaction between the spin label and the protein may be exothermic or endothermic. The labeling of protein with Cu­(II)-NTA is typically performed at lower temperatures as the reaction is exothermic.^78^ We, therefore, analyzed the effect at three different temperatures (0, 4, and 25 °C). Over the assessed temperature range, we do not observe a marked influence on protein binding as they all yielded a similar percentage of component 1 (cf. Figure S3B).

Together, these data indicate that while Cu­(II)-TPA successfully binds to the dHis site, the label does not bind stoichiometrically. Therefore, we determined the effect of the Cu­(II)-TPA:dHis ratio on protein binding. This is a significant consideration as excess Cu­(II)-TPA in solution can contribute to the background signal in dipolar spectroscopy, leading to the loss of sensitivity in measurements. The amount of the dHis-bound component as a function of the ratio of Cu­(II)-TPA to dHis was obtained by simulation of the CW spectra. The amount of the dHis-bound component increases to 70% as the label:dHis ratio reaches 1.25:1 but then begins to decrease as excess Cu­(II)-TPA in the solution increases. These results are encouraging as the Cu­(II)-TPA complex seems to support octahedral dHis coordination as desired, and the labeling is achieved within 10 min. However, 100% labeling was not attained under the conditions we tested.

To further confirm successful coordination of the label to histidine sites on the protein, we conducted X-band pulsed electron spin echo envelope modulation (ESEEM).^79^ This experiment measures the interaction between the Cu­(II) center and the distally noncoordinating nitrogen of histidine. For E15H/T17H/K28H/Q32H GB1, ESEEM was performed at two field positions indicated by vertical lines in the simulated field-swept spectrum in the inset of Figure 3A. The first field corresponds to the maximum of the FS-ESE spectrum where the protein-bound component is 65–70%. However, there are contributions from the free label at this field position. At a lower field, only the protein-bound component contributes to the signal. A control experiment with Cu­(II)-TPA was also performed. Figure 3A shows the ESEEM signal of the protein at the two fields, 3300 and 2878 G in blue and black, respectively. The data show modulations typically seen with His coordination. The ESEEM signal for free Cu­(II)-TPA, colored gray, does not show any modulations because TPA does not contain a remote nitrogen. Figure 3B shows the ESEEM spectra of the protein at the two fields. Both spectra show three nuclear quadrupolar interaction peaks below 2 MHz and a broad double quantum peak at ∼4 MHz that are characteristic of Cu­(II) coordinating to histidine.^80–87^ The nuclear quadrupolar interaction peaks are very well resolved for the spectrum at the low field, suggesting a homogeneous coordination between the label and dHis protein. The peak at a higher frequency (i.e., at around 12 and 14 MHz for the two data sets) corresponds to ^1^H ESEEM. The intensity of the double quantum peak at the maximum field is lower than the intensity observed in the spectrum at 2878 G. This is expected because at the maximum field, there are contributions from the free Cu­(II)-TPA component.

**Figure 3:**
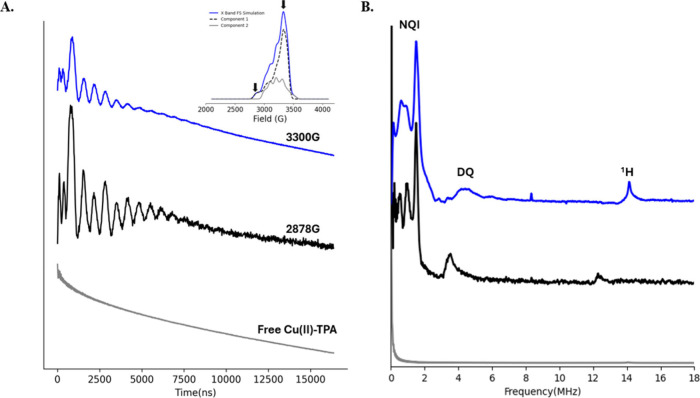
(A) Time domain ESEEM signal for free Cu­(II)-TPA (gray) or Cu­(II)-TPA bound to E15H/T17H/K28H/Q32H GB1 at fields of 2878 G (black) and 3300 G (blue). The inset shows the simulated FS-ESE spectrum at X-band. The spectra of the bound component (component 1) and the free component (component 2) are shown with a dashed black line and gray line, respectively. The arrows indicate the two magnetic fields at which the ESEEM experiment was performed. (B) ESEEM spectra are shown. Overall the data suggest that Cu­(II)-TPA coordinates to dHis GB1.

Since the ESEEM at 2878 G is solely from the protein-bound component, we compared the spectrum to the ESEEM spectrum of a complex containing Cu­(II) coordinated to two imidazole rings. Figure S5 shows the two ESEEM spectra. The relative intensity of the double quantum peak versus the single quantum peak and the normalized intensity of the ^14^N peaks to ^1^H peaks were comparable for both samples. Together, these data suggest that the Cu­(II) binds to both His residues.^83,85,87,88^

To probe orientational selectivity, we performed DEER at Q-band on two different mutants of GB1: 15H/17H/28H/32H and 6H/8H/28H/32H GB1-labeled Cu­(II)-TPA. The data were collected at four field positions across the field-swept spectrum. The vertical lines on the FS-ESE spectrum in the inset of Figure 4B denote the pump pulse positions for the measurements. For each of these measurements, the observer frequency was set to a value 300 MHz higher than that of the pump. The background-subtracted time domain signals for the 15H/17H/28H/32H construct are shown in Figure 4A. The primary data are provided in Figure S6A, and the experimental parameters are detailed in Table S2. The frequency of the modulation does not change with magnetic field. Even at a field of 11 300 G where the free label component contributes ∼50%, the dipolar modulations are preserved. A time trace resulting from the summation of the four fields after normalizing to their relative intensity is shown in black in Figure 4A. Indeed, the summed time trace also shows the same period of modulation. These data signify that there is minimal orientational selectivity with Cu­(II)-TPA at Q-band frequencies. Figure 4B shows the distance distributions at different fields. The distributions show the expected distance of 2.2 nm for this mutant of GB1 and are similar across the fields. The data were also analyzed using DEERNet^89^ and yield similar results (cf. Figure S7). We prepared a fresh batch of protein, and data on this replicate also confirm proper orientation averaging (cf. Figure S8). We have also performed in silico simulations according to a previously described procedure^63,64^ to analyze orientational sampling at Q-band. The simulation data and details are provided in Figure S9.

**Figure 4:**
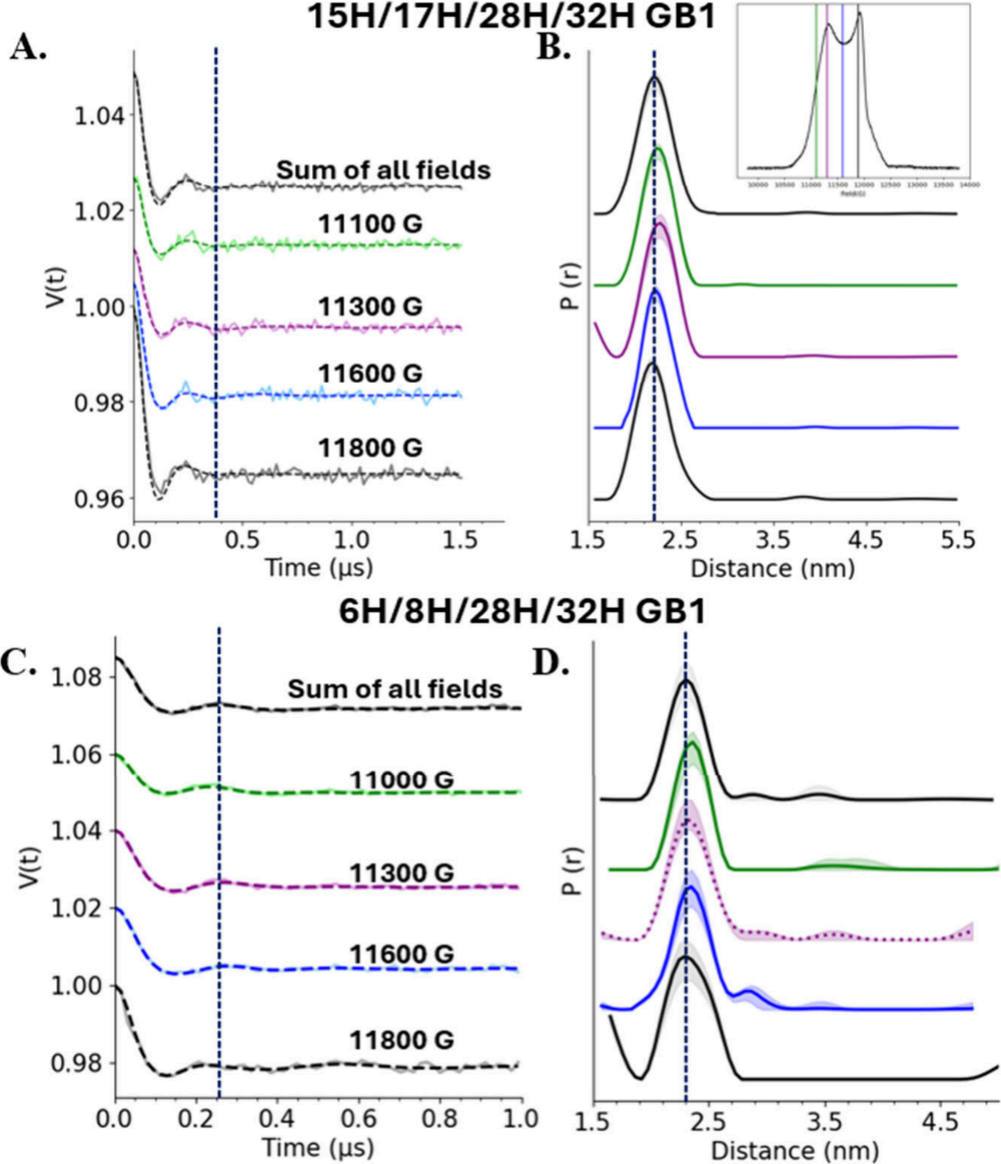
(A) Background-subtracted and normalized time domain signal obtained at the different fields for 15H/17H/28H/32H GB1. The sum of the time traces obtained at the four fields is colored black at the top. The dashed line shows that the modulation frequency is consistent across the fields. (B) Distance distributions for 15H/17H/28H/32H GB1 obtained from DeerAnalysis via Tikhonov regularization at the different field positions. The most probable distance is 2.2 nm and is unchanged among all of the magnetic field positions. The shaded areas show uncertainty in distance analysis. (C) Background-subtracted time traces for the 6H/8H/28H/32H mutant of GB1 across different field positions. (D) Corresponding distance distributions that are largely similar across fields.

Figure 4C shows the background-subtracted time traces for 6H/8H/28H/32H GB1 acquired across the different fields. The primary data and experimental parameters are given in Figure S6B and Table S3, respectively. The period of modulation is consistent across the fields and even for the summed trace. Figure 4D shows the corresponding distance distribution for this mutant. The distribution is centered at around 2.3 nm and is largely similar across fields. This result is especially important because previous work using this mutant of GB1 labeled with Cu­(II)-NTA showed orientational selectivity at Q-band.^49^

Additionally, we also prepared six samples (15H/17H/28H/32H) at higher concentrations and different labeling conditions and performed DEER at X-band, where orientation effects are anticipated to be minimal. The data are shown in Figure S11. The distance distributions obtained at X-band were identical to those obtained at Q-band (Figure S12A), which further validates that a single field acquisition at Q-band is sufficient for orientational averaging.

This result is interesting because the use of Cu­(II)-TPA as a spin label narrows the breadth of the FS-ESE spectrum by only ∼17% (1800 G for Cu­(II)-NTA vs 1500 G for Cu­(II)-TPA). A distinguishing feature of the Cu­(II) labels is that they exhibit sufficient affinity for the dHis site^77,78^ but do not bind tightly.^90^ Thus, the lengths and angles of the bond that coordinate the Cu­(II) to the imidazole ring of His have a range of values that lead to a range of orientations of the **g** tensor axes.^43,91^ Such orientational flexibility largely reduces the effect of orientational selectivity such that data with Cu­(II)-NTA can usually be obtained at one field at X-band.^41,43,90,92^ For Q-band DEER, a computational strategy was recently developed to decipher orientational effects in Cu­(II)-NTA.^62^ Based on the insights, acquisition schemes that involve experiments at only three fields^63^ or two fields^64^ were established and provided orientation-independent distance measurements. Nevertheless, both approaches require one or more data collections at low magnetic fields where the echo size is small. In this work, the data suggest that the slight reduction of the spectral width for Cu­(II)-TPA seems to be sufficient to wash out orientation effects at Q-band such that a single field collection is adequate. These Cu­(II) labels are thus distinct from tightly bound Cu­(II) systems that can show orientation selectivity in DEER even at X-band.^93,94^ It is also instructive to compare the distribution width obtained with the use of Cu­(II)-TPA to examine the precision of the label. Figure S12B illustrates the distribution width obtained with the Cu­(II)-NTA spin label in contrast to Cu­(II)-TPA. The distance is centered around 2.3 nm for both labels, which is consistent with the predicted distance from other modeling techniques.^95,96^ The standard deviation of the distance distribution for Cu­(II)-TPA is 1.8 Å compared to 1.2 Å for Cu­(II)-NTA.^40,77^

To decipher whether the label coordinates to single-His sites, we performed additional experiments. Control DEER experiments on a single-His construct (15H/28H) of GB1 showed a 6-fold reduction in modulation depth and no discernible modulation consistent with a distance of 2.2 nm. In addition, DEER experiments with 15H/17H/28H/32H GB1 in the presence of free imidazole further suggest that Cu­(II)-TPA prefers dHis coordination. These data are shown in Figures S13 and S14, respectively.

Finally, to compare Cu­(II)-TPA and Cu­(II)-NTA in terms of sensitivity, we labeled 100 μM tetramutant GB1 with Cu­(II)-NTA and performed DEER at Q-band. Primary data and experimental parameters are given in Figure S17 and Table S5. For Cu­(II)-NTA, at least two acquisitions are required to obtain orientationally averaged distances.^62,64^ The inset of Figure 5A shows the two pump positions overlaid on the FS-ESE spectrum of Cu­(II)-NTA-labeled GB1. The data were acquired for four scans at each field, which took approximately 2 h at each field position. Figure 5A shows the background-subtracted time trace for the summed data for the two fields. We observed a modulation depth, λ, of 2.2% for the summed data, and the signal-to-noise ratio was found to be 45 after acquisition for ca. 4 h. For Cu­(II)-NTA, a low-field acquisition (803 G lower than the main field) close to the *g*_∥_ region is necessary to sufficiently obtain an orientationally averaged distance measurement. The echo size here is roughly 2.2 times smaller than the main field. In addition, the modulation depth is also much lower. The need to acquire data at this low-field position compromises the sensitivity of Cu­(II)-NTA.

**Figure 5:**
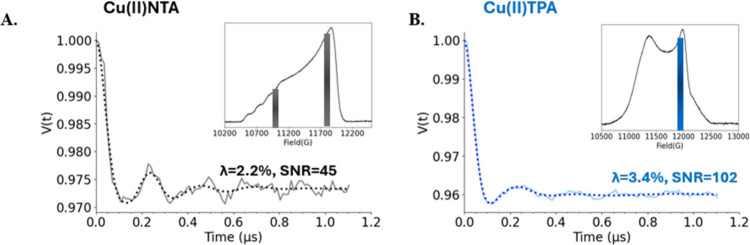
(A) Background-subtracted DEER signal using Cu­(II)-NTA label. The signal was constructed by summing the time domain signals obtained at two fields. The inset shows the echo-detected field-swept spectrum for Cu­(II)-NTA, indicating the field positions of the pump pulses in the two experiments. (B) Background-subtracted DEER time trace using the Cu­(II)-TPA label. The data was acquired at the field position of maximum intensity. The inset shows the FS-ESE spectrum for Cu­(II)-TPA depicting the position of the pump pulse.

Next, DEER was performed on the protein sample labeled with Cu­(II)-TPA. The data were acquired at the maximum of the echo-detected field-swept spectrum shown in the inset of Figure 5B. Figure 5B shows the background-subtracted time domain signal. We achieve a λ of 3.4% as the data were collected at the field position of maximum intensity. Furthermore, the signal-to-noise ratio was 102 after data acquisition for approximately 2 h. Notably, these data suggest that with Cu­(II)-TPA there is a ca. 2.3-fold improvement in sensitivity in half the acquisition time despite the lower labeling efficiency.

In looking at the future, the use of Cu­(II)-TPA as a spin label offers a significant benefit over Cu­(II)-NTA, as one can potentially achieve proper orientational averaging at the magnetic field of maximum intensity at Q-band. In turn, such an acquisition ensures a higher sensitivity and reduces data collection times. Additionally, unlike Cu­(II)-NTA, free Cu­(II)-TPA exhibits a different **g** tensor compared to that of its protein-bound form. This is reflected in the features of the FS-ESE spectrum collected at Q-band (cf. Figure S18). At the magnetic field of maximum intensity, there is an only ∼20% contribution from the free label. As a result, a small excess of Cu­(II)-TPA can be added to ensure labeling without substantially compromising the sensitivity. This is not the case with Cu­(II)-NTA where free and bound labels both have an octahedral coordination geometry. Therefore, there is considerable overlap between their spectra. However, the TPA label exhibits lower affinity for dHis sites as compared to existing Cu­(II)-based labels. In this work, we were able to achieve only about 65–70% labeling. The low solubility of TPA in water due to its hydrophobicity is a challenge for labeling. The addition of small amounts of cosolvents such as acetonitrile resulted in only minor improvements in labeling efficiency. Thus, further work that introduces hydrophilic substituents into TPA may be valuable. In addition, it would also be important to assess if the label binds to single-histidine sites on a protein in the presence of dHis.^44^ This needs to be examined carefully on several proteins with native single-His sites as was done for Cu­(II)-NTA.^44,45,97^ This is not limiting as the creation of a Cys-null (or in this case His-null) background is the standard protocol for site-directed spin labeling. Further improvements in the sensitivity may be realized by the use of time-variable RIDME measurements. Finally, generating force field parameters to combine EPR with molecular dynamics simulations^90,97^ and developing analysis software^95,96^ would be useful to promote the use of this label.

In summary, we have developed a new Cu­(II) spin label for proteins. The spin label is created by coordination of Cu­(II) to tris­(2-pyridylmethyl)­amine. In this scheme, central Cu­(II) is chelated to four nitrogen atoms, which helps reduce *g* anisotropy. We show that the label binds to the proposed double-histidine sites on the protein using CW-EPR and ESEEM experiments. Due to the low solubility of TPA in water, labeling efficiencies of ca. 70–80% were achieved. Nevertheless, the spin label provides accurate and narrow distance distributions. Remarkably, the Q-band DEER data in different fields exhibit the same modulation period. This result provides a significant benefit because it suggests that one acquisition at the maximum of the field-swept electron spin echo-detected spectrum can sample all orientations of the interspin vector. The use of this label promises significant gains in signal sensitivity, despite incomplete labeling.

## Supplementary Material



## Notes

Protein expression and purification protocols and experimental methods, primary CW-EPR and simulations, labeling pH and temperature optimization, primary DEER data and parameters, X-band DEER, comparison of distribution widths, control DEER experiments, and analysis using DEERNet and simulated FS-ESE (PDF)
