# Supplementary material for: Rational Design of a Cu(II) Spin Label Improves the Sensitivity of Distance Measurements
Source: J Phys Chem Lett. 2025 Sep 26;16(39):10256–64. doi: 10.1021/acs.jpclett.5c02221 (PMC12498500; doi:10.1021/acs.jpclett.5c02221)
Supplement: Supplementary file 1 [file jz5c02221_si_001.pdf]

# Supporting Information

# Rational Design of a Cu(II) Spin Label Improves Sensitivity of Distance Measurements.

*Shramana Palit, Zikri Hasanbasri, Nicholas A. Moriglioni, Joshua Casto and Sunil Saxena**

Department of Chemistry
University of Pittsburgh
219 Parkman Ave. Pittsburgh PA 15260

**Corresponding Author**

*Sunil Saxena - Department of Chemistry, University of Pittsburgh, Pittsburgh. PA 15260, USA.
ORCID: 0000-0001-9098-6114, Phone (412) 624-8680. Email: sksaxena@pitt.edu.

METHODS

*Sample Preparation*

Five dHis mutants of of B1 immunoglobulin binding domain of protein G (GB1) were used in this work E15H/T17H, K28H/Q32H,E15H/T17H/K28H/Q32H, I6H/N8H/T17H/K28H and E15H/K28H. Previous studies have shown that double histidine mutation does not significantly impact the structure or the melting temperature of the protein.[1] BL21 (DE3) E. coli cells were purchased from New England Biolabs. The GB1 mutant plasmid was purchased from Synbio Technologies. Mutagenesis, expression, and purification of protein were done according to previously published methods.[2,3] The protein concentration ranged from 50μM to 250 μM. TPA was purchased from Tokyo Chemicals Industry. Labeling methods with Cu(II)-TPA are described in the results and discussions section. The X-Band sample volumes were 120 μL and the Q Band sample volume was 20 μL. The protein samples were prepared in 50 mM HEPES buffer with and without AcN and at two pH conditions-6.5 and 7.4. 20% glycerol was added as a cryoprotectant for CW and ESEEM samples. The DEER samples were prepared in 40% deuterated glycerol to extend phase memory time. Each sample was flash frozen using precooled propylene + propane (MAP-Pro).[4]

*EPR Measurements*

X-Band experiments were conducted using a Bruker ElexSys E680 X-band FT/CW spectrometer equipped with a Bruker ER4118X-MD5 resonator. The temperature for all experiments was controlled using an Oxford ITC503 temperature controller with an Oxford ER 4118CF gas flow cryostat. CW-EPR experiments were performed at X Band (~9.68 GHz) at 80K. Data was collected for 1024 points with a sweep width of 2000G centered at 3100 G. All CW EPR experiments used a modulation amplitude of 4 G, modulation frequency of 100 kHz and a conversion time of 20.48 ms at an attenuation of 30 dB. The CW simulations were done using EasySpin[5] software.

Three-pulse ESEEM experiments were performed at X Band at a temperature of 18 K.[6] A $\pi/2$– $\tau$– $\pi/2$– T– $\pi/2$– echo pulse sequence was used. The $\pi/2$ pulse length was set to be 10 ns. ESEEM was performed at two fields- the magnetic field with the maximum intensity and a lower field around 2800 G. At the maximum field, $\tau$ was set to 132 ns and the initial value of T was 264 ns and at the lower field they were set to be 152 ns and 304 ns. The value of T was stepped by a step size 16 ns for a total of 1024 steps. The resultant signal was Fourier-transformed using Bruker WinEPR software.

DEER experiments were carried out at both X Band and Q Band at 18K. A four-pulse DEER pulse sequence was used: $(\pi/2)\nu 1-\tau 1-(\pi)\nu 1-T-(\pi)\nu 2-\tau 2-(\pi)\nu 1-\tau 2$ echo. For the X Band measurements, observer $\pi/2$ and $\pi$ pulse of 10 and 20 ns were used respectively and the pump pulse was 16 ns. The data was collected over 100 points with a step size of 14 ns. The pump frequency and the observer frequency had a 100 MHz offset. The shot repetition time was set to 1500 μs and the shots per point was set to 20. The experimental time domain signal was analyzed by DeerAnalysis 2022b which uses Tikhonov Regularization.[7]

For Q Band distance measurements, Bruker ElexSys FT/CW spectrometer with a Bridge12 resonator, Bruker SpinJet AWG and a 300 W amplifier were used. Observer pulses of 6 and 12 ns were used. For the data in the main text, the pump pulse was a frequency-swept Chirp pulse of 48 ns with a frequency ranging from -300 to -100 MHz relative to the observer frequency. The data was collected over 132 points with a step size of 14 ns. For the replicate measurement at Q Band, a CHIRP pulse of 48 ns was used. The data was collected for 125 points with a step size of 14 ns.

For sensitivity comparison experiments, observer pulses of 8 and 16 ns were used. The pump pulse was a frequency-swept CHIRP pulse of 64 ns with frequencies ranging from -400 to -150 MHz relative to the observer frequency. The data was collected over 128 points with a step size of 12 ns. For Cu(II)-TPA, the DEER was performed at the maximum intensity of the FS-ESE spectrum. For Cu(II)-NTA, two measurements were done with the pump placed -124G and -803G away from the maximum. The resultant data was summed to account for orientational selectivity.

*Easy Spin Simulations:*

The CW-EPR spectra were simulated using EasySpin 5.2.36 toolbox in MATLAB[5] with the Pepper program. The microwave frequency, center field, sweep width, no. of points and temperature were fixed according to the experimental parameters. The spectra were simulated using two components by varying the g and A tensors of both components to obtain a fit for the experimental data. The weights of the two components were also varied to obtain the percentage of labeled protein. A-strain and g-strain were kept constant at 0.

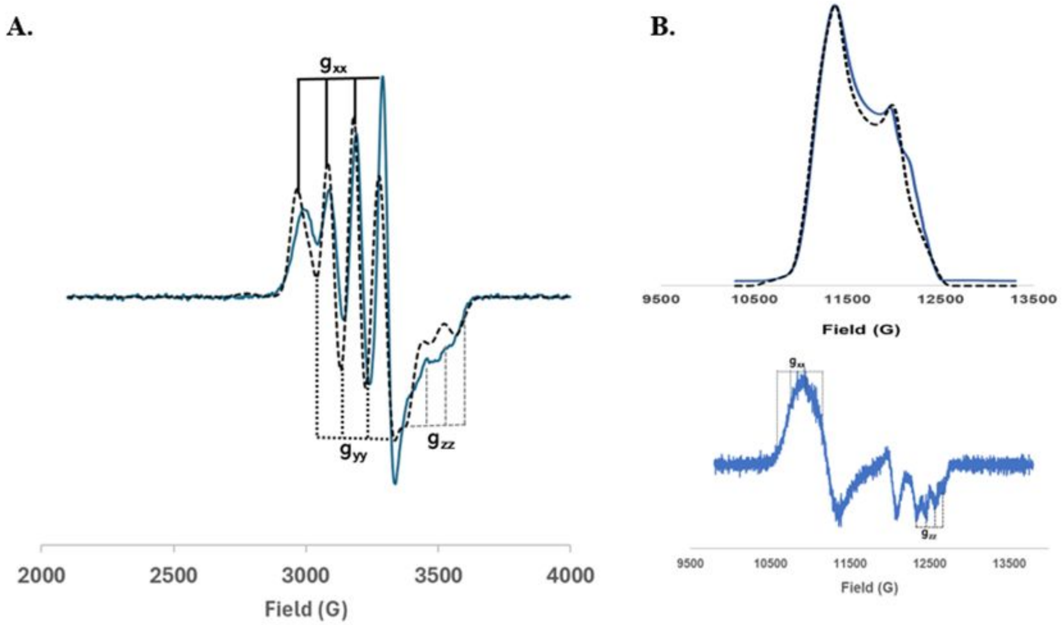


**Figure S1**. A) Experimental CW EPR spectrum of Cu(II)-TPA obtained at X Band in blue. The simulated spectrum is shown in black dashed lines using one component B) (Top) The echo detected field swept spectrum of Cu(II)-TPA obtained at Q band in blue and the simulated spectrum is shown in dashed lines. (Bottom) The first derivative of the FS-ESE spectrum at Q-Band which is analogous to a CW-EPR spectrum obtained at Q-Band. The $g_{zz}$ and $A_{zz}$ region of the spectra is resolved. The simulations at X and Q-Band taken together suggest that $g_{xx}$=2.199, $g_{yy}$=2.188 and $g_{zz}$=2.030, and $A_{xx}$=113 G, $A_{yy}$=75 G and $A_{zz=}$98 G for this complex.

To determine the labeling efficiency on single dHis sites on the protein we prepared 28H/32H and 15H/17H mutants of GB1. The CW-EPR spectra and the simulations on these mutants spin labeled with Cu(II)-TPA are shown in Figure S2. The bound component was 80±5% for the alpha helical site and 70±3% for the beta sheet. . Based on the percent labeling of single sites, the overall labeling efficiency of doubly bound GB1 is calculated to be 56±4%.

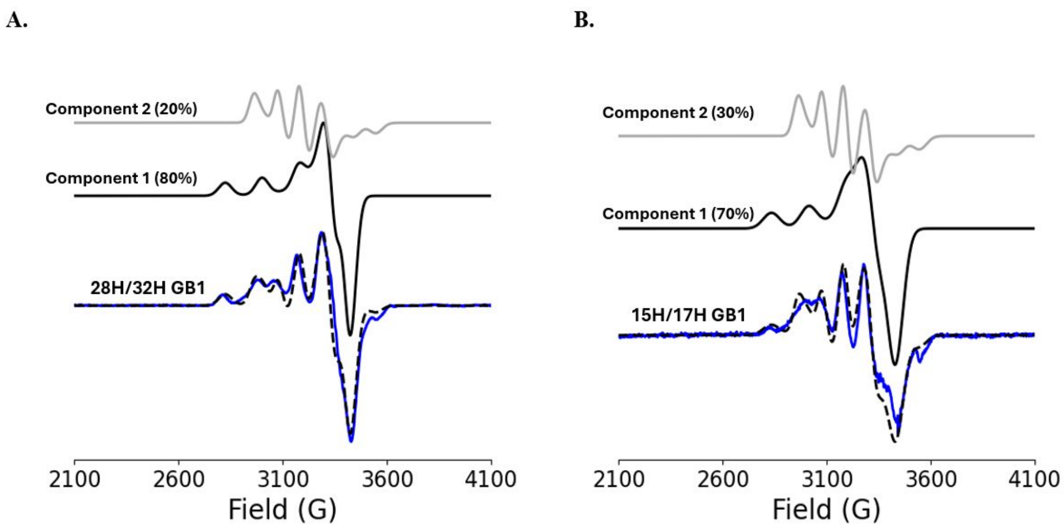


**Figure S2.** A) Experimental (blue) and simulated (black dashed) CW-EPR spectrum of 28H/32H bound Cu(II)-TPA indicating a two-component fit. Component 1 (80%) is indicative of an octahedral coordination arising from Cu(II)-TPA coordinating to two histidine sites. The component arising from free Cu(II)-TPA in solution is shown in light grey. B) Experimental (blue) and simulated spectrum for 15H/17H GB1. The bound component is ~70% for this beta sheet site.

**Table S1**: g and A tensors for the simulated spectrum of Cu(II)-TPA bound to protein.

| | $g_{xx}$ | $g_{yy}$ | $g_{zz}$ | $A_{xx}$ | $A_{yy}$ | $A_{zz}$ |
|---|---|---|---|---|---|---|
| Bound Component | 2.070 | 2.070 | 2.231 | 10 G | 10 G | 171 G |
| Free Label | 2.199 | 2.188 | 2.030 | 113 G | 75 G | 98 G |

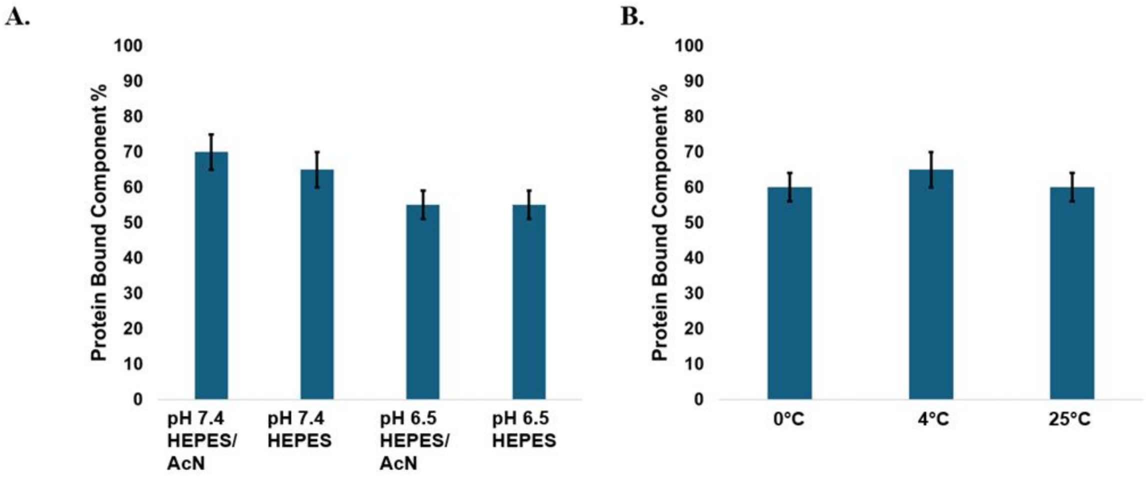


**Figure S3**. Bar graph showing the variation of the amount of dHis bound to Cu(II)-TPA with A) different pH and buffer conditions; and *B*) incubation temperatures. To obtain the optimal binding conditions for Cu(II)-TPA to protein, we tested two pH conditions and a range of incubation temperatures- 0˚C, 4˚C and 25˚C. We also added a small amount to acetonitrile to analyze its effect on labeling as the ligand TPA has low solubility in water. We obtained CW-EPR spectrum for each of these samples and simulated the spectrum to obtain the percent of octahedral component. The error bars are calculated by changing the component ratio in EasySpin till the simulated spectrum changes significantly. Primary CW EPR spectra are shown in Figure S3.

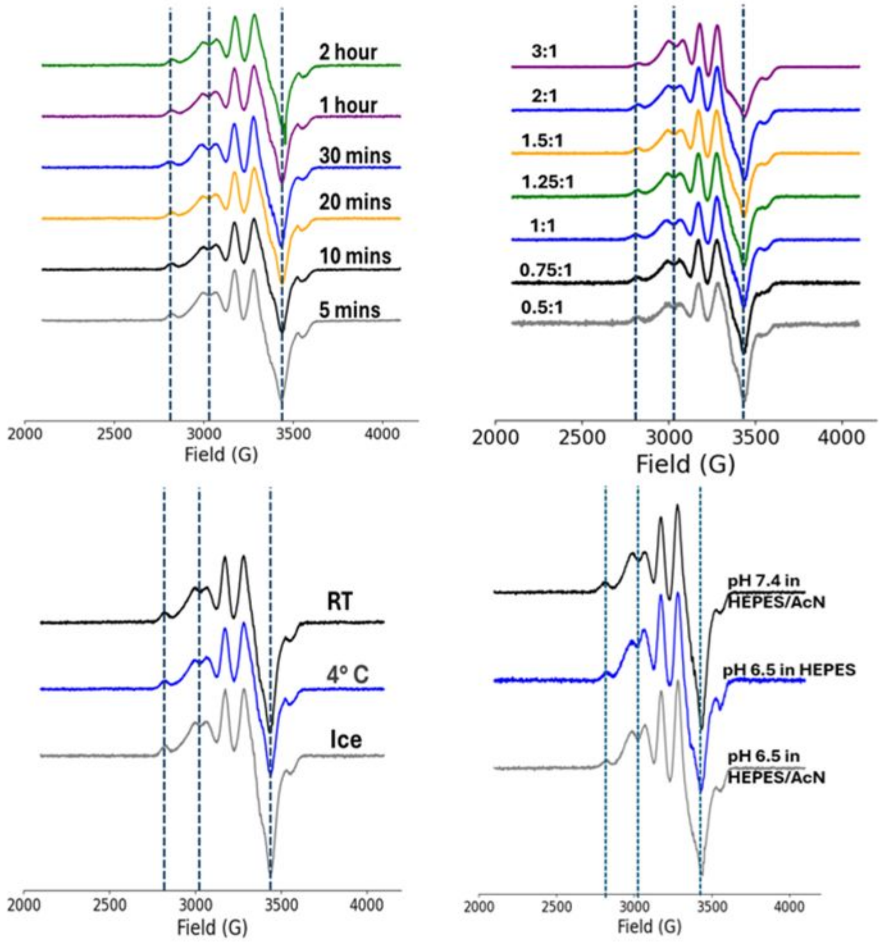


Figure S4. Primary CW-EPR spectra for the different labeling optimization experiments. The CW spectra were simulated to obtain the percentage of protein bound label. The dashed lines indicate the appearance of the octahedral component arising from protein loading.

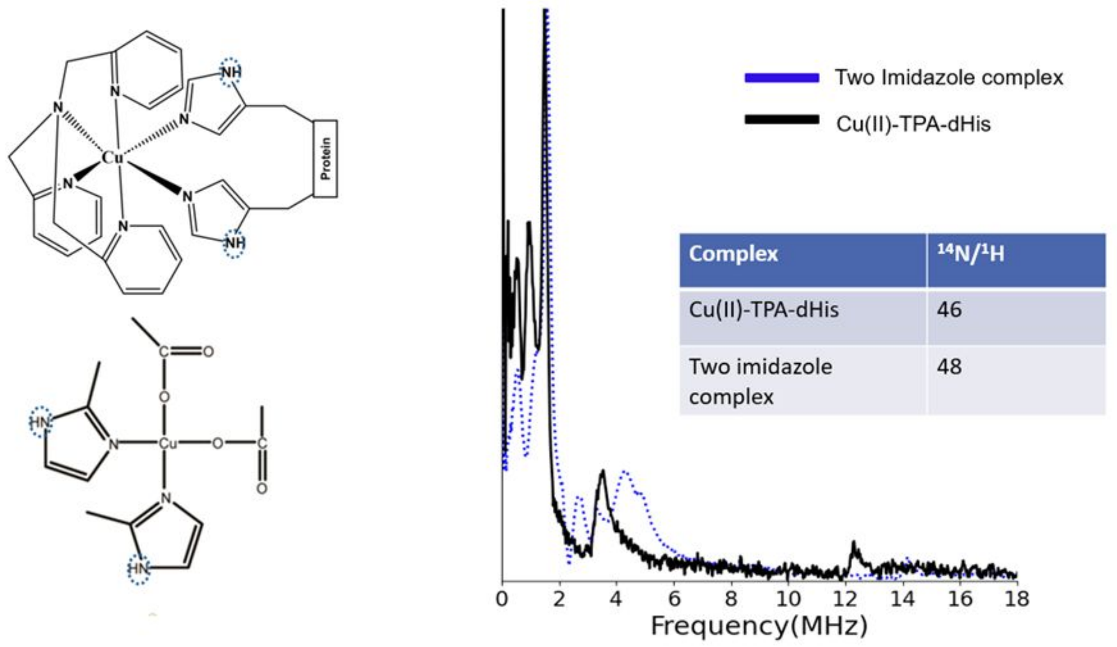


**Figure S5**. Structures of bis(2-methylimidazole)copper and dHis protein coordinated Cu(II)-TPA (left). The distally coordinated nitrogen that can be detected through ESEEM is circled. Overlayed ESEEM spectra of dHis protein labeled with Cu(II)-TPA at the low field position (black) and of a Cu(II) complex where the metal ion is coordinated to two imidazole rings (blue). The frequency peaks below 2 MHz (nuclear quadrupolar interaction) and the broad peak around 4 MHz (double quantum). The intensity of the double quantum peak relative to the nuclear quadrupolar interaction peak increases with an increase in the number of histidine coordinated. To quantify the number of histidines coordinated to Cu(II)-TPA, the ratio of the normalized intensity of $^{14}$N to $^{1}$H was taken. The normalized integrated intensity ratio of $^{14}$N to $^{1}$H of Cu(II)-TPA-dHis was found to be 46, whereas for the two imidazole complex it was 48. Such comparison relies on the fact that proton contribution in these surface sites is largely due to solvent and thus the proton peak can serve as an internal standard to account for different signal to noise ratios.[8–11]

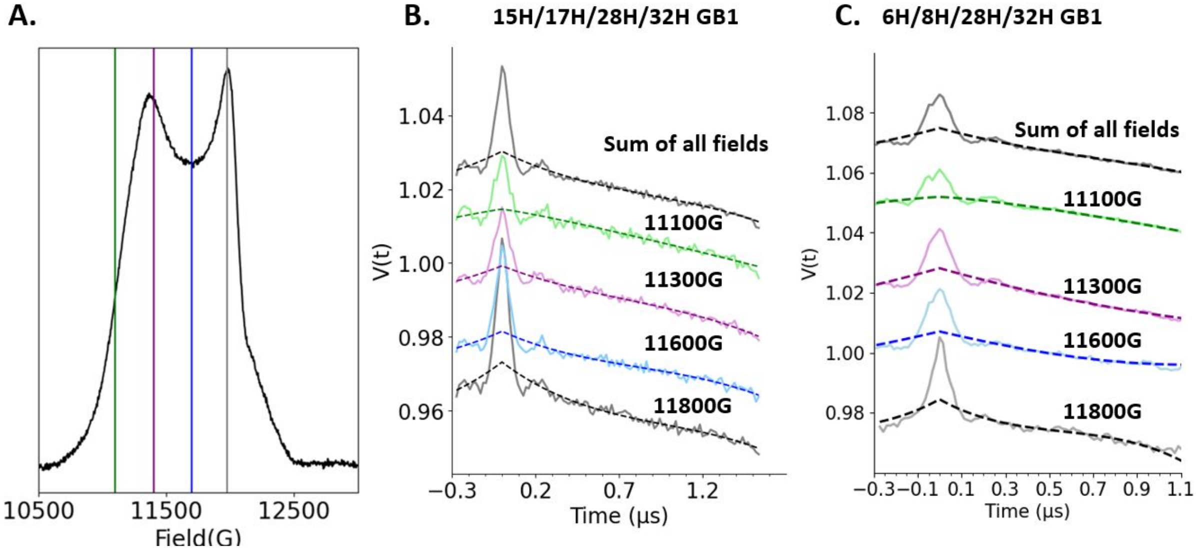


Figure S6. Primary Q-Band DEER data (main text). A) FS-ESE of GB1 labeled with Cu(II)-TPA at Q-Band with vertical lines indicating the pump pulse positions. B) Primary DEER time traces for the data presented in the main text for 15H/17H/28H/32H GB1 at different field positions. C) Primary DEER time traces for the data presented in the main text for 6H/8H/28H/32H GB1 at different field positions.

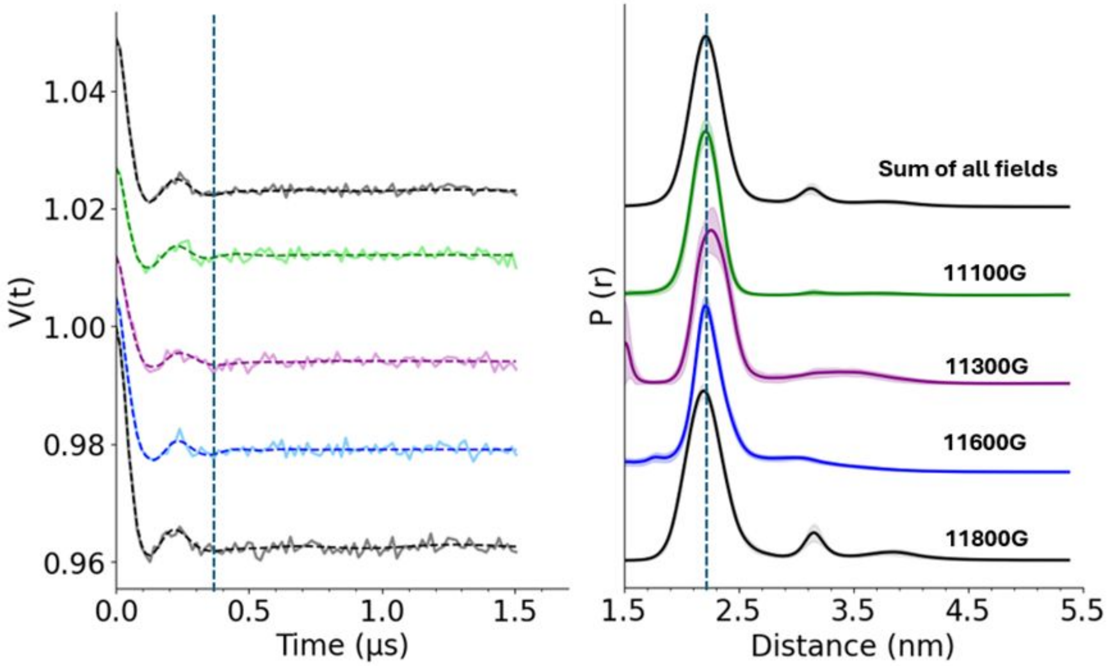


**Figure S7**. The Q-Band data for 15H/17H/28H/32H GB1was also analyzed by DEERNet which is a one-step analysis procedure. A) Background subtracted time domain signal with the fits obtained plotted in dashed lines. For each of these traces, the frequency of modulation remains the same therefore yielding the same results as DEERAnalysis via Tikhonov Regularisation.[7] B) Distance distributions from DEERNet. They are largely similar across the different fields, suggesting proper orientational averaging.

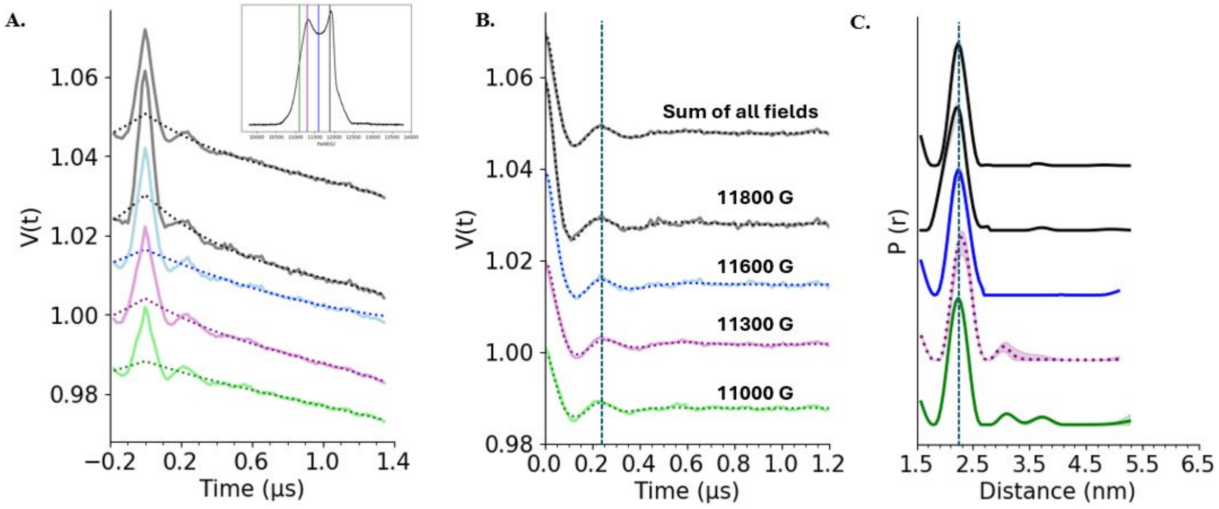


**Figure S8**. Biological replication of the Q-Band data for 15H/17H/28H/32H GB1. A fresh batch of protein was prepared and spin labeled to repeat the measurements across the different magnetic field positions at Q-Band. A) Primary DEER time traces acquired across different field positions. The inset shows the FS-ESE spectrum with vertical lines indicating the pump pulse positions of the DEER. B) Background subtracted time traces with the fits obtained from DEERAnalysis.[12] The modulation period of the traces obtained at different fields is the same indicating small orientational selectivity effects at Q-Band. C) The corresponding distance distributions.

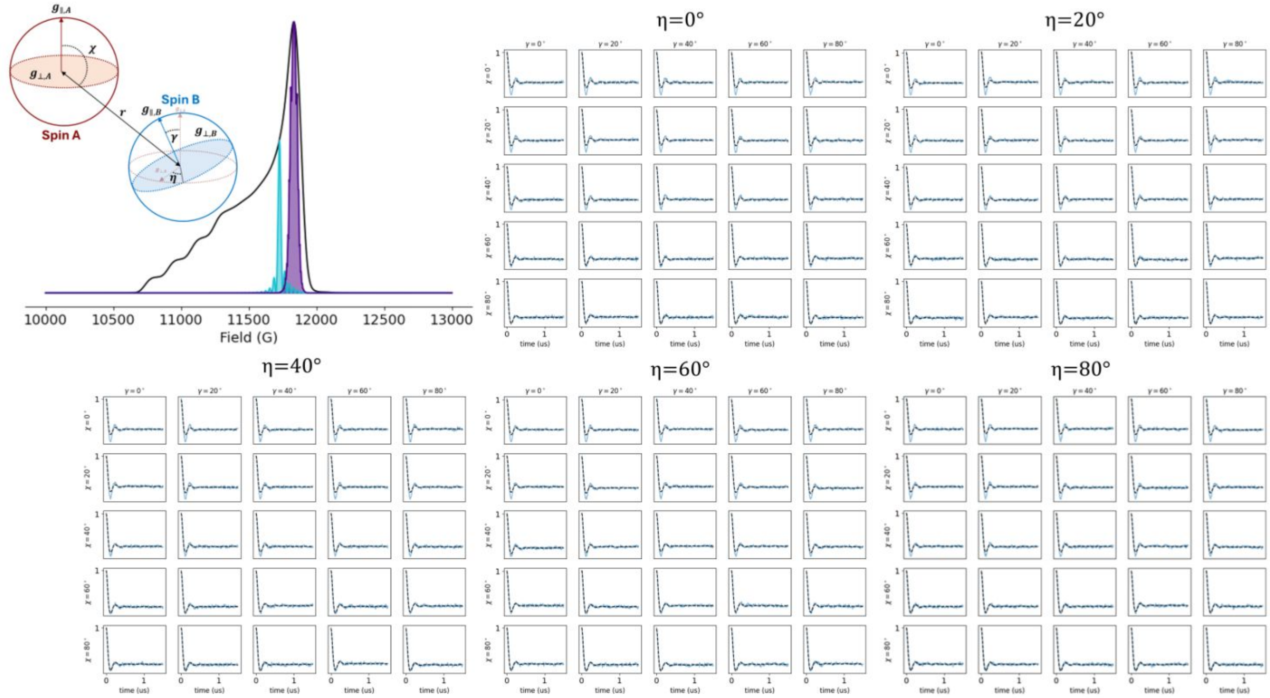


**Figure S9.** Simulated Q-band FS-ESE spectrum (top left) for the protein-bound component of Cu(II)-TPA. The g- and hf tensors measured experimentally were used for these simulations. The simulated FS-ESE is produced by summing the line shapes produced by a statistical distribution of randomly oriented spins. Simulated data (right) comparing the DEER traces for complete excitation and selective excitation using experimental parameters.

In order to analyze sampling, we also performed simulations according to previously described procedures. [13,14] Figure S9 (top left) shows the protein-bound component of the FS-ESE at Q-Band for Cu(II)-TPA. The pump and probe pulses used for the DEER experiment are overlaid on the FS-ESE spectrum. Figure S9 shows the simulated data comparing the DEER signals obtained under conditions of complete spin excitation (black dashed) with those expected from selective excitation using the experimental pulses (blue). In the simulations, the relative orientations of the g-tensors are described using three angles ($\chi$, $\gamma$, and $\eta$). For this system there exists a distribution of relative orientations in the sample and a key contributor to washing out the orientation effects in DEER is the standard deviation of the relative orientation. The larger the distribution width the lower is orientation selectivity in DEER.[13,14] Orientations were tested systematically by changing

the mean value of each of the three angles $\chi, \gamma, \& \eta$ from 0° to 80° in steps of 20°. Since the standard deviations for the Cu(II)-TPA label are not available we used the standard deviation values that were estimated for the Cu(II)-NTA label.[15] The S.D. values for $\chi, \gamma, \& \eta$ was set to be 28°, 25° and 72° respectively. Given that the Cu(II)-TPA label is more flexible than the Cu(II)-NTA label, these values are likely underestimates. Thus, simulations were performed for 125 different relative orientations. Post simulation noise was added to obtain an SNR of 50:1. As is seen from Figure S9, the simulated traces are mostly identical to full excitation except for the angles where χ is small. However, even for those relative orientations, the modulation frequency is largely preserved.

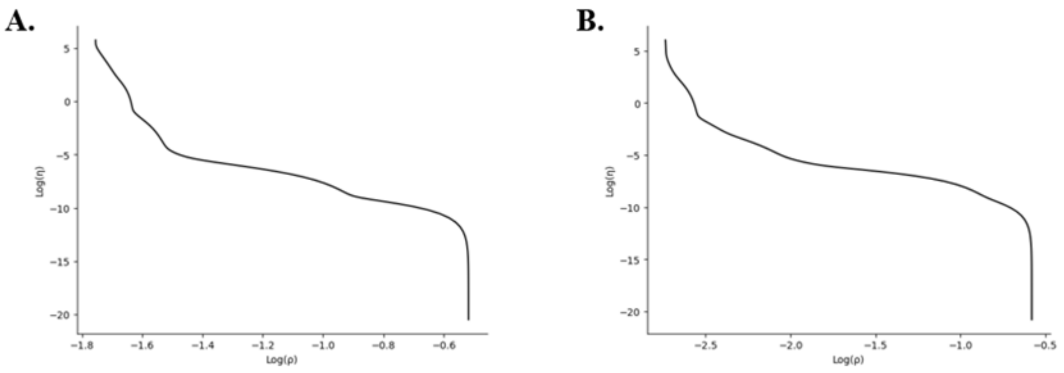


**Figure S10**. L-curves obtained from DEERAnalysis via Tikhonov Regularization for A) summed trace of all fields in Figure 4 in the main text and B) the data for sensitivity comparison in Figure 5.

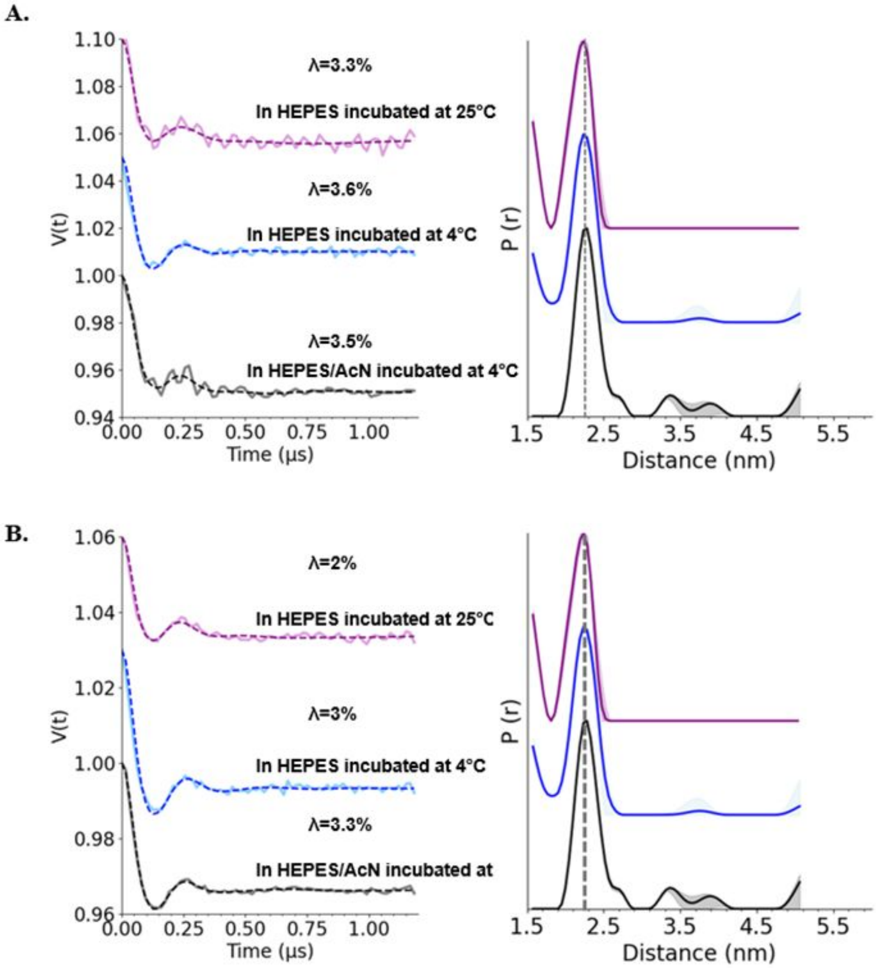


**Figure S11**. Background subtracted and normalized time traces obtained at X Band for 250 μM 15H/17H/28H/32H GB1 labeled with 500 μM Cu(II)-TPA obtained at A) pH 7.4 and B) 6.5 in different labeling conditions. The amount of AcN was less than 7.5% of the total sample volume. The modulation depth, λ, is indicated for each experiment. Distance distributions obtained from the time domain signals are shown on the right. Distance is centered around 2.2 nm which is the expected distance for this GB1 construct. The X-Band data shows that the label can provide accurate and narrow distance constraints. The modulation depth obtained for the measurements at both pH conditions was lower than that observed with Cu(II)-NTA due to lower labeling efficiency.

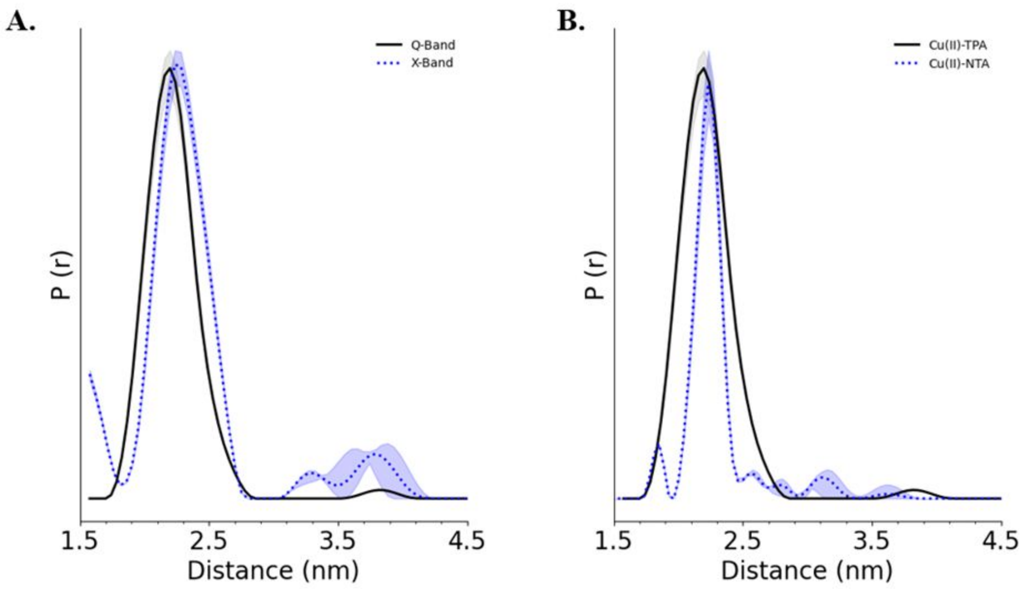


**Figure S12**. A) Distance distributions obtained with 15H/17H/28H/32H GB1 labeled with Cu(II)-TPA at X-Band (blue) and Q-Band (black). The distributions are identical, indicating proper orientational averaging at one magnetic field at Q-Band. B) Comparison of distribution widths obtained with Cu(II)-TPA (black) and Cu(II)-NTA. The S.D. of the distribution obtained with Cu(II)-TPA is 1.8 Å, while for Cu(II)-NTA it is 1.2 Å.

As a control we also prepared a single His mutant of GB1 (E15H/K28H) with a His residue at two sites. ESEEM experiment on this mutant shows that Cu(II)-TPA does bind to the single His sites. The ESSEM spectrum is shown in Figure S13A. This is not unexpected since Cu(II)-NTA also coordinates to single His sites – however, it preferentially binds to dHis sites on the protein if they are present.[15] Next, we performed DEER on 15H/28H GB1. The DEER trace is shown in Figure S13B (top) in comparison to the DEER trace on dHis mutant (bottom). The modulation depth was ca. six times smaller than that observed with E15H/T17H/K28H/Q32H GB1. The DEER data of 15H/28H GB1 also lacked distinct modulation corresponding to the expected 2.2 nm distance and the modulation depth was too low (0.6%) to extract any distance. This data suggests that while Cu(II)-TPA can bind to single His sites, the binding efficiency to single sites is significantly lower than dHis sites. Secondly, this data further confirms that Cu(II)-TPA supports dHis coordination since we measured the anticipated distance only from the dHis mutant.

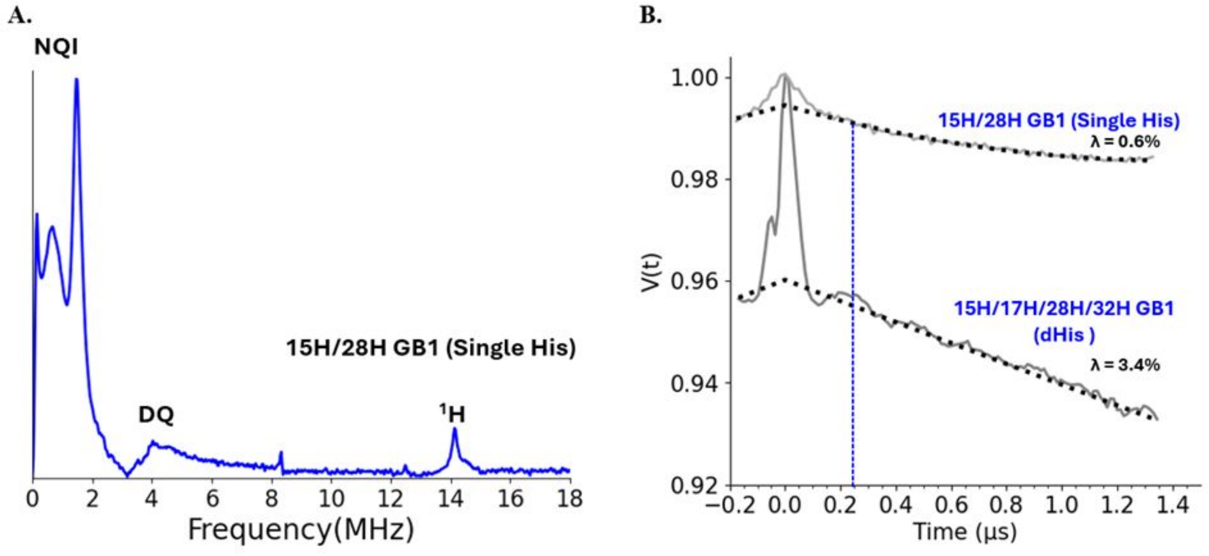


**Figure S13.** A) ESEEM spectrum of 15H/28H GB1 labeled with Cu(II)-TPA. There are characteristic NQI and DQ peaks which are present with histidine coordination indicating that Cu(II)-TPA coordinates to these

single His sites on the protein. B) DEER trace on the single mutant compared to dHis mutant. The DEER on the single mutant lacks distinct modulations and has a very low modulation depth.

Additionally, to test whether Cu(II)-TPA preferentially coordinates to single His rather than dHis sites on the protein, we performed DEER measurements on 100 μM dHis-GB1 labeled with 200 μM Cu(II)-TPA in the presence of 100 μM free imidazole. The background subtracted time domain signal on this sample is shown in Figure S14. The modulation depth remains unchanged relative to samples without excess imidazole, indicating that the coordination of the label to the dHis site is stable even in the presence of competing imidazole. Cu(II)-TPA preferentially coordinates to dHis sites on the protein.

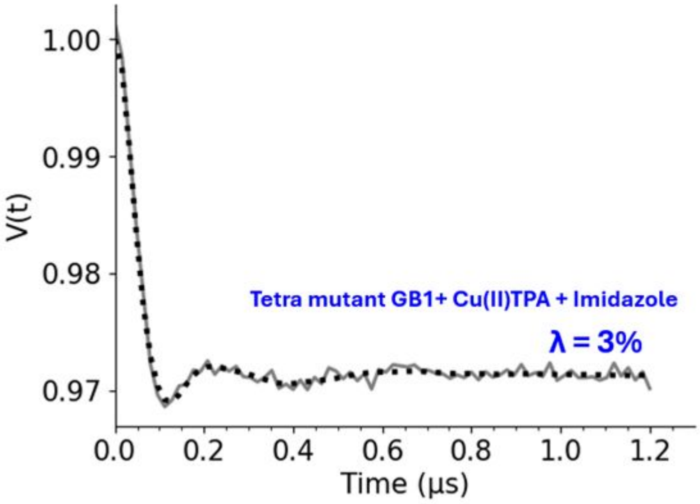


**Figure S14.** Background subtracted time domain signal for tetramutant GB1 labeled with Cu(II)-TPA in presence of free imidazole.

Finally, data on a mutant with a single dHis site (28H/32H) GB1 shows no modulations. This data is shown in Figure S14.

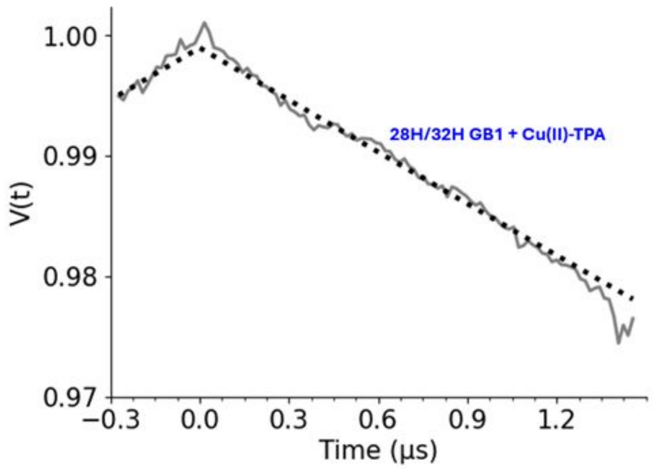


**Figure S15.** DEER on singly labeled GB1 (28H/32H). There are no modulations as expected.

**Table S2.** DEER acquisition parameters, modulation depths and SNR, for Cu(II)-TPA labeled 15H/17H/28H/32H GB1 at different field positions. (*data in main text*)

| Field | 11800 G | 11600 G | 11300 G | 11100G | SUM |
|---|---|---|---|---|---|
| $(\pi/2)\upsilon_A$ (ns) | 6 | 6 | 6 | 6 | 6 |
| $(\pi)\upsilon_A$ (ns) | 12 | 12 | 12 | 12 | 12 |
| $(\pi)\ \upsilon_B$ | 48 ns 200 MHz chirp | 48 ns 200 MHz chirp | 48 ns 200 MHz chirp | 48 ns 200 MHz chirp | 48 ns 200 MHz chirp |
| $\tau$ | 400 | 400 | 400 | 400 | 400 |
| T | 1600 | 1600 | 1600 | 1600 | 1600 |
| $\Delta t$ | 14 | 14 | 14 | 14 | 14 |
| SRT (ms) | 1.5 | 1.5 | 1.5 | 1.5 | 1.5 |
| Shots per point | 20 | 20 | 20 | 20 | 20 |
| No. of averages | 64 | 90 | 78 | 99 | |
| Modulation depth % | 3.0 | 2.4 | 1.6 | 1.4 | 2.4 |
| SNR [b] | 48 | 35 | 30 | 23 | 48 |

[b] SNR was calculated based on modulation depth and determined directly from SNR Calculator.

**Table S3.** DEER acquisition parameters, modulation depths and SNR, for Cu(II)-TPA labeled 6H/8H/28H/32H GB1 at different field positions. (*data in main text*)

| Field | 11800 G | 11600 G | 11300 G | 11100G | SUM |
|---|---|---|---|---|---|
| $(\pi/2)\upsilon_A$ (ns) | 6 | 6 | 6 | 6 | 6 |
| $(\pi)\upsilon_A$ (ns) | 12 | 12 | 12 | 12 | 12 |
| $(\pi)\ \upsilon_B$ | 48 ns 200 MHz chirp | 48 ns 200 MHz chirp | 48 ns 200 MHz chirp | 48 ns 200 MHz chirp | 48 ns 200 MHz chirp |
| $\tau$ | 400 | 400 | 400 | 400 | 400 |
| T | 1400 | 1400 | 1400 | 1400 | 1400 |
| $\Delta t$ | 14 | 14 | 14 | 14 | 14 |
| SRT (ms) | 1.5 | 1.5 | 1.5 | 1.5 | 1.5 |
| Shots per point | 20 | 20 | 20 | 20 | 20 |
| No. of averages | 15 | 35 | 88 | 112 | |
| Modulation depth % | 2 | 1.6 | 1.5 | 1 | 1.3 |
| SNR [b] | 37 | 52 | 60 | 42 | 56 |

[b] SNR was calculated based on modulation depth and determined directly from SNR Calculator.

**Table S4.** DEER acquisition parameters, modulation depths and SNR, for biological and technical repeat. (*data in SI*)

| Field | 11800 G | 11600 G | 11300 G | 11100G | SUM |
|---|---|---|---|---|---|
| $(\pi/2)\upsilon_A$ (ns) | 6 | 6 | 6 | 6 | 6 |
| $(\pi)\upsilon_A$ (ns) | 12 | 12 | 12 | 12 | 12 |
| $(\pi)\ \upsilon_B$ | 48 ns 200 MHz chirp | 48 ns 200 MHz chirp | 48 ns 200 MHz chirp | 48 ns 200 MHz chirp | 48 ns 200 MHz chirp |
| $\tau$ | 400 | 400 | 400 | 400 | 400 |
| T | 1600 | 1600 | 1600 | 1600 | 1600 |
| $\Delta t$ | 14 | 14 | 14 | 14 | 14 |
| SRT (ms) | 1.5 | 1.5 | 1.5 | 1.5 | 1.5 |
| Shots per point | 20 | 20 | 20 | 20 | 20 |
| No. of averages | 54 | 98 | 155 | 180 | |
| Modulation depth % | 3.2 | 2.5 | 1.8 | 1.4 | 2.5 |
| SNR [b] | 67 | 91 | 118 | 77 | 90 |

[b] SNR was calculated based on modulation depth and determined directly from SNR Calculator.

**Table S5**. DEER acquisition parameters, modulation depths and SNR for Q Band DEER for comparison of sensitivity between Cu(II)-NTA and Cu(II)-TPA

| Label | Cu(II)-NTA | Cu(II)-TPA |
|---|---|---|
| $(\pi/2)\upsilon_A$ (ns) | 10 | 10 |
| $(\pi)\upsilon_A$ (ns) | 20 | 20 |
| $(\pi)\ \upsilon_B$ | 64 ns 250 MHz chirp | 64 ns 250 MHz chirp |
| $\tau$ | 400 | 400 |
| T | 1800 | 1800 |
| $\Delta t$ | 12 | 12 |
| SRT (ms) | 1.5 | 1.5 |
| Shots per point | 20 | 20 |
| No. of averages | 8, 8 | 8 |
| Modulation depth % | 2.2 | 3.4 |
| SNR | 45 | 104 |

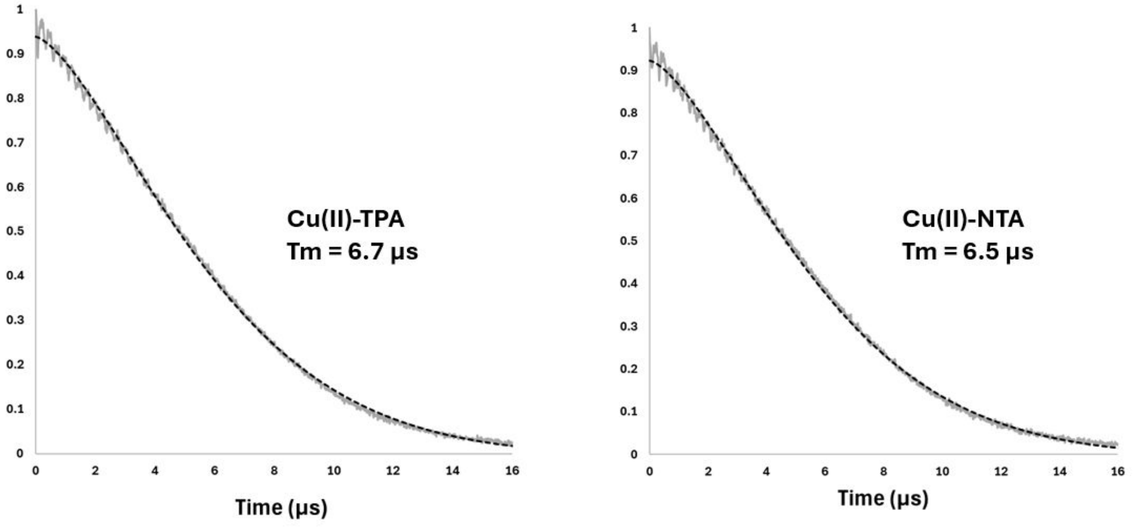


**Figure S16**. The echo decay data for protein labeled Cu(II)-TPA (left) and Cu(II)-NTA (right) in deuterated solvent and deuterated glycerol. The data was for with a stretched exponential function on XEPR and the Tm values are comparable between the two samples.

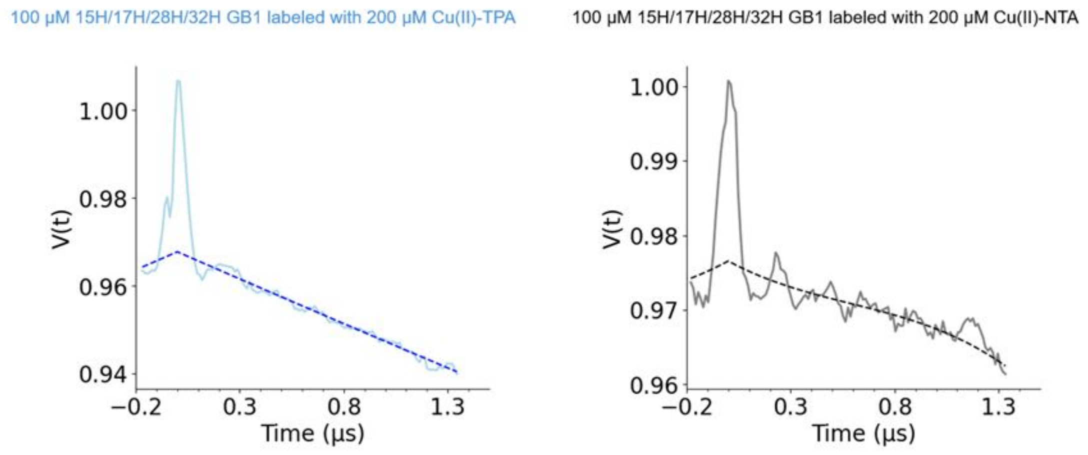


**Figure S17**: Primary DEER time domain signals for Cu(II)-TPA labeled GB1 (black) and Cu(II)-NTA labeled GB1 (blue) shown in solid lines. The background is plotted in dashed lines. DEER parameters are detailed in Table S5.

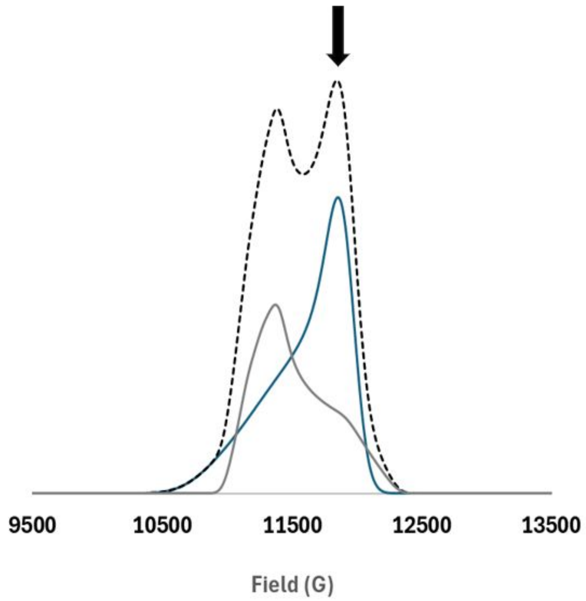


**Figure S18**. Simulated total FS-ESE spectrum shown in dashed black. The two components- protein bound (blue) and free label (grey) are overlaid. The arrow indicates the magnetic field position of maximum intensity of the bound component. It is evident that there is 20% contribution from the free label at this position and thus, small excess Cu(II)-TPA can be added to ensure labeling without substantially compromising sensitivity.


(1) Cunningham, T. F.; Putterman, M. R.; Desai, A.; Horne, W. S.; Saxena, S. The Double-Histidine Cu2+-Binding Motif: A Highly Rigid, Site-Specific Spin Probe for Electron Spin Resonance Distance Measurements. *Angew. Chem. Int. Ed.* **2015**, *54* (21), 6330–6334. https://doi.org/10.1002/anie.201501968.

(2) Cunningham, T. F.; McGoff, M. S.; Sengupta, I.; Jaroniec, C. P.; Horne, W. S.; Saxena, S. High-Resolution Structure of a Protein Spin-Label in a Solvent-Exposed β-Sheet and Comparison with DEER Spectroscopy. *Biochemistry* **2012**, *51* (32), 6350–6359. https://doi.org/10.1021/bi300328w.

(3) Cunningham, T. F.; Pornsuwan, S.; Horne, W. S.; Saxena, S. Rotameric Preferences of a Protein Spin Label at Edge-Strand β-Sheet Sites. *Protein Sci.* **2016**, *25* (5), 1049–1060. https://doi.org/10.1002/pro.2918.

(4) Singewald, K.; Wilkinson, J. A.; Saxena, S. Copper Based Site-Directed Spin Labeling of Proteins for Use in Pulsed and Continuous Wave EPR Spectroscopy. *Bio-Protoc.* **2021**, *11* (24). https://doi.org/10.21769/BioProtoc.4258.

(5) Stoll, S.; Schweiger, A. EasySpin, a Comprehensive Software Package for Spectral Simulation and Analysis in EPR. *J. Magn. Reson.* **2006**, *178* (1), 42–55. https://doi.org/10.1016/j.jmr.2005.08.013.

(6) Casto, J.; Bogetti, X.; Hunter, H. R.; Hasanbasri, Z.; Saxena, S. “Store-Bought Is Fine”: Sensitivity Considerations Using Shaped Pulses for DEER Measurements on Cu(II) Labels. *J. Magn. Reson.* **2023**, *349*, 107413. https://doi.org/10.1016/j.jmr.2023.107413.
(7) Jeschke, G.; Chechik, V.; Ionita, P.; Godt, A.; Zimmermann, H.; Banham, J. E.; Timmel, C. R.; Hilger, D.; Jung, H. DeerAnalysis2006 –A Comprehensive Software Package for Analyzing Pulsed ELDOR Data. *Appl Magn Reson V30 473-498 2007* **2006**, *30*. https://doi.org/10.1007/BF03166213.
(8) Shin, B.; Saxena, S. Substantial Contribution of the Two Imidazole Rings of the His13−His14 Dyad to Cu(II) Binding in Amyloid-β(1−16) at Physiological pH and Its Significance. *J. Phys. Chem. A* **2011**, *115* (34), 9590–9602. https://doi.org/10.1021/jp200379m.
(9) Silva, K. I.; Michael, B. C.; Geib, S. J.; Saxena, S. ESEEM Analysis of Multi-Histidine Cu(II)-Coordination in Model Complexes, Peptides, and Amyloid-β. *J. Phys. Chem. B* **2014**, *118* (30), 8935–8944. https://doi.org/10.1021/jp500767n.
(10) Goldfarb, D.; Fauth, J. M.; Tor, Y.; Shanzer, A. Study of Copper(II) Binding to Chiral Tripodal Ligands by Electron Spin Echo Spectroscopy. *J. Am. Chem. Soc.* **1991**, *113* (6), 1941–1948. https://doi.org/10.1021/ja00006a012.
(11) Shin, B.; Saxena, S. Direct Evidence That All Three Histidine Residues Coordinate to Cu(II) in Amyloid-B1−16. *Biochemistry* **2008**, *47* (35), 9117–9123. https://doi.org/10.1021/bi801014x.
(12) Seal, M.; Feintuch, A.; Goldfarb, D. The Effect of Spin-Lattice Relaxation on DEER Background Decay. *J. Magn. Reson.* **2022**, *345*, 107327. https://doi.org/10.1016/j.jmr.2022.107327.
(13) Bogetti, X.; Hasanbasri, Z.; Hunter, H. R.; Saxena, S. An Optimal Acquisition Scheme for Q-Band EPR Distance Measurements Using $Cu^{2+}$-Based Protein Labels. *Phys. Chem. Chem. Phys.* **2022**, *24* (24), 14727–14739. https://doi.org/10.1039/D2CP01032A.
(14) Hasanbasri, Z.; Moriglioni, N. A.; Saxena, S. Efficient Sampling of Molecular Orientations for Cu(II)-Based DEER on Protein Labels. *Phys. Chem. Chem. Phys.* **2023**, *25* (19), 13275–13288. https://doi.org/10.1039/D3CP00404J.
(15) Heubach, C. A.; Hasanbasri, Z.; Abdullin, D.; Reuter, A.; Korzekwa, B.; Saxena, S.; Schiemann, O. Differentiating between Label and Protein Conformers in Pulsed Dipolar EPR Spectroscopy with the dHis-Cu2+(NTA) Motif. *Chem. – Eur. J.* **2023**, *29* (72), e202302541. https://doi.org/10.1002/chem.202302541.
